# Estimating density of forest bats and their long‐term trends in a climate refuge

**DOI:** 10.1002/ece3.10215

**Published:** 2023-06-17

**Authors:** Bradley Law, Traecey Brassil, Roland Proud, Joanne Potts

**Affiliations:** ^1^ Forest Science Unit NSW Primary Industries Parramatta New South Wales Australia; ^2^ Cupar Analytics Ltd Cupar UK; ^3^ Analytical Edge Statistical Consulting Blackmans Bay Tasmania Australia

**Keywords:** banding, climate refuge, density, drought, elevation, mark–recapture, old growth, timber harvesting

## Abstract

For many species, estimating density is challenging, but it is important for conservation planning and understanding the functional role of species. Bats play key ecological roles, yet little is known about their free‐ranging density. We used a long‐term banding study of four species caught in an extensively forested climate refuge and spatial capture–recapture models (SCR) to estimate density and its change over time. Between 1999 and 2020, there were 3671 captures of four bat species, which were all edge‐space foragers. Recaptures represented 16% (*n* = 587) of all captures, of which 89 were between‐trap‐cluster movements. Closed spatial mark–recapture models estimated plausible densities that varied with elevation. Preferred elevations differed between species, with density averaging 0.63 ha^−1^ for *Vespadelus darlingtoni* (high elevation), 0.43 ha^−1^ for *V. pumilus* (low elevation), 0.19 ha^−1^ for *Chalinolobus morio* (high elevation), and 0.08 ha^−1^ for *V. regulus* (high elevation). Overall, densities were higher than most previous published estimates for bats. Forest disturbance history (past timber harvesting) had no detectable effect on density. Density also varied substantially across years, and although annual maximum temperature and rainfall were not supported in models, some time periods showed an apparent relationship between density and annual rainfall (+ve) and/or annual maximum temperature (−ve). The most notable change was an increase in the density of *V. pumilus* after 2013, which tracked an increase in annual temperature at the site, reflecting a warming climate. Bat densities in forests outside of climate refugia are likely to be more sensitive to climate change, but more studies are needed in different habitats and continents and outside climate refugia to place the densities we estimated into a broader context.

## Graphical abstract

A long‐term banding study of four bat species caught in an extensively forested climate refuge was used with spatial capture–recapture models to estimate species density and changes over time. Models estimated plausible densities that varied with elevation and across years. Our results suggest that bat densities in productive forests may be substantially higher than previously appreciated with variation over time inconsistently related to climate patterns.

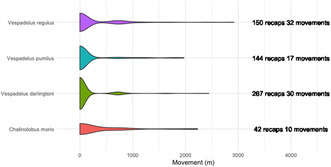

## 1 INTRODUCTION

Density and abundance are key attributes of animal populations of interest to the general public and land managers (Caughley & Sinclair, 1994; Denes et al., 2015). Basic knowledge of how many individuals occur in an area is important for assessing and informing conservation decisions (Mace et al., 2008), assisting in interpreting the functional role of species in an ecosystem, be they predators or prey (Lacher et al., 2019; Sundstrom et al., 2018), and estimating the number of animals impacted by major disturbances such as megafires (Van Eeden et al., 2020). Yet, density and other attributes are especially difficult or expensive to estimate for mobile or cryptic species. Nonetheless, there have been pleas for more rigorous attempts at estimating rather than guessing density (Hone & Buckmaster, 2015).

Bats are mobile and cryptic taxa that are highly diverse with recent evidence revealing that they can play functionally important roles in many ecosystems (Boyles et al., 2013; Kolkert et al., 2021). Inferences about functional importance depend on knowledge of species abundance or density, which has traditionally been difficult to derive for forest species that roost in tree hollows. Indeed, there are very few estimates available for bat density and current global comparisons suggest that bats occur at lower density than terrestrial or arboreal mammals (Santini et al., 2022). Existing estimates mostly derive from extrapolating from counts in day roosts over their presumed nightly foraging area (Dwyer, 1966; Jones et al., 1996) or via modeled relationships of roost counts with habitat variables (Clement & Castleberry, 2013). For example, a minimum population density of 0.13 individuals ha^−1^ was estimated for *Pipistrellus pipistrellus*, based on counts at summer (maternity) roosts in buildings dispersed across a broad region of northern England (Jones et al., 1996). More recent estimates have been based on random encounter modeling of the calling activity of unmarked individuals across acoustic arrays (Milchram et al., 2020). In this case, low‐density estimates were derived from calling activity in upland spruce forests of Germany, which estimated average densities of 0.003 ha^−1^ for *Eptesicus nilssonii* (open space forager), 0.003 ha^−1^ for *Myotis nattereri* (closed space forager), and 0.06 ha^−1^ for *P. pipistrellus* (high frequency, edge‐space forager). These studies were undertaken in cool temperate climate zones of Europe, but very different densities could be expected in other climate zones or other habitats, potentially driven by productivity and/or anthropogenic disturbance (Brawn et al., 2001; Carbone & Gittleman, 2002; Hamer et al., 2022; Stephens et al., 2019). For example, density of birds and mammals is typically high in temperate wet areas with intermediate levels of productivity (Santini et al., 2018). Both approaches for estimating density have assumptions related to nightly foraging area that is difficult to define and estimates of density will be sensitive to the accuracy of that area, while calling rate may also vary temporally.

Spatial capture–recapture (SCR) models are an alternative contemporary approach for estimating density based on recaptures of marked animals from multiple known locations (Borchers & Efford, 2008; Royle & Young, 2008), but they have not been applied to bat populations. SCR models provide spatially robust estimates of animal density using information in the spatial pattern of recapture events (relative to where traps were set) to model a capture function (Borchers & Efford, 2008; Royle et al., 2013). The capture function is conceptually consistent with a detection function in distance sampling (Buckland et al., 2004), whereby the probability of detecting an animal is a monotonically declining function of the distance between an animal's home range center and the known “trap” locations at which the animal might be detected (a “trap” being defined as any detector such as a physical trap like the harp traps used here). These models have not been used to develop estimates of bat density, but they could provide an important contribution to improving the available density estimates for this functionally important group.

We used a long‐term banding study based on trapping at multiple sites in montane forest and SCR to estimate density of four edge‐space foraging, vespertilionid bats. In addition to our aim of using SCR to model density, annual banding over 20 years since 1999 provided us with the opportunity to assess changes in density over time within this montane climate refuge. Banding spanned a period of increasing warmer climate reflecting global climate change (Bellard et al., 2015) as well as wet and dry periods, including the Australian “Millennium drought” (1997–2010) (Verdon‐Kidd & Kiem, 2009) and another severe drought that led to megafires in the Australian summer of 2019–2020 (although the study area did not burn). Survival analysis based on the first 14 years of these banding data revealed a large portion of the population remained resident, with a maximum time to recapture of 9 years (Law et al., 2018). Apparent survival of residents was not strongly influenced by weather variation (except for the smallest species), probably because the wet, montane forest provided a climate refuge (Law et al., 2018). Recruitment was not recorded, but annual variation could lead to fluctuating density. We predicted that density would be relatively stable over time due to the study location in a climate refuge and the longevity of bats. We also expected bat density would be higher than previously published estimates because our study area was dominated by productive, tall forests that were extensive, although with some timber harvesting.

## 2 MATERIALS AND METHODS

The study area (734 ha) fell within an experimental section (450–940 m elevation) of Chichester State Forest, 200 km north of Sydney, Australia (32°09′53″ S, 151°42′11″ E). The experimental section, which comprises eight small (13–97 ha) catchments, was established in 1974/75 to investigate the effects of logging on water flow and quality (see Cornish, 1993; Law et al., 2018). The area is surrounded by extensive forests that are at different stages of regeneration following timber harvesting as well as being adjacent to Barrington Tops National Park. Regrowth in the study area originated from timber harvesting in 1983 for the hydrology experiment, with two catchments left unlogged (Cornish, 1993).

The area mainly comprises tall wet sclerophyll forest (35 m). Dominant tree species included Sydney blue gum (*Eucalyptus saligna*) (43%), silvertop stringybark (*E. laevopinea*) (23%), and pure and mixed rainforest communities (32%), where crabapple (*Schizomeria ovata*), sassafras (*Doryphora sassafras*), and corkwood (*Quintinia sieberi*) were the primary species (Cornish & Vertessy, 2001). Creek lines are dominated by rainforest species, which also contributed considerably to understory composition across the study area.

### 2.1 Study species

The four most commonly captured species were the subject of analyses. The species varied in body mass: chocolate‐wattled bat *Chalinolobus morio* (8 g), large forest bat *Vespadelus darlingtoni* (6 g), southern forest bat *Vespadelus regulus* (5 g), and eastern forest bat *Vespadelus pumilus* (4 g). Each belongs to the Vespertilionidae family and forages in edge spaces, usually within forested communities in south‐eastern Australia.

### 2.2 Bat sampling

Six to nine (three additional sites were added in 2013) permanent trapping clusters were sampled annually in autumn (~4 months after parturition) over 21 years (1999–2020). We standardized trapping effort at each site using a cluster of one to three harp traps set within a 200 m length along a 4WD trail. We attempted to keep the precise location of traps consistent between years, but locations sometimes varied slightly due to track maintenance. In each trap year, we trapped for two consecutive nights at each cluster to minimize trap avoidance, except that occasionally banding trips were repeated when rain affected trapping. The first 2 years of the study were an exception, in that three trapping events were employed in autumn (separated by ~ 2 weeks) for the purpose of maximizing the number of bands in the population for recapture and survival analysis in later years (see Law et al., 2018). Positions of individual traps were changed slightly (<100 m) within a cluster from year to year due to changes in vegetation on flyways, where bats are trapped. A central location was selected on GIS for each cluster of two to three traps from 1999 to 2016, and from 2017 to 2020, individual trap locations were recorded with a GPS to more precisely document bat movements at a local scale (both between clusters and individual traps) to explore whether this improved the precision of estimates in these years. Mean distance between cluster centroids was 1681 m (range = 599–2998 m).

**FIGURE 1:**
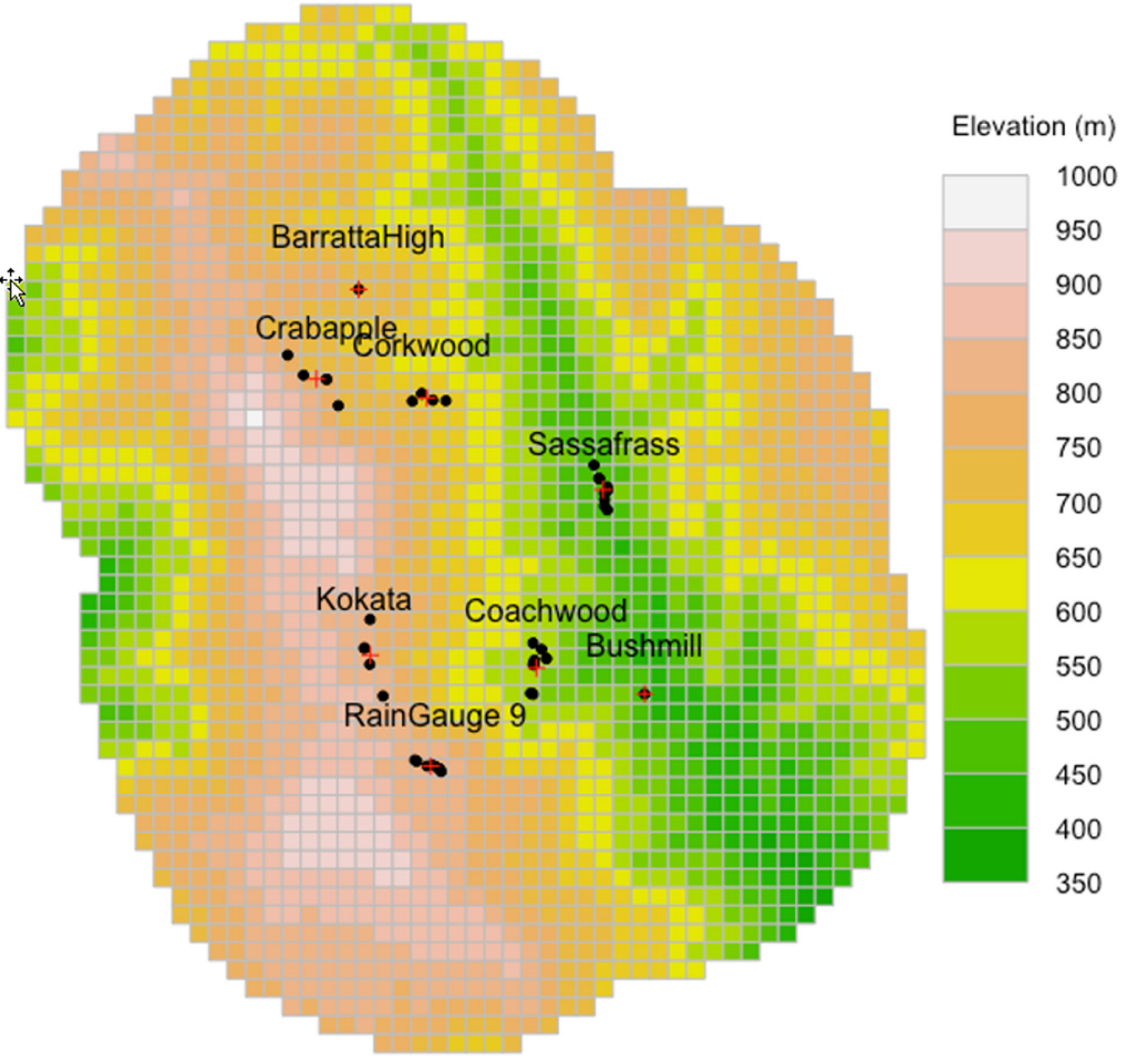
Elevation mapped to the study area mask with trap locations (black points) and cluster name and centroid (red cross). Note that elevation was categorized for analysis as high (>600 m) or low (<600 m).

Each trap cluster sampled two adjacent forest catchments of the same disturbance history, thus extending the small spatial scale of the individual catchments in the original hydrology experiment to account for the large area requirements of bats (Cornish, 1993). Disturbance history averaged 78 ha in area, with old‐growth forest (including rainforest) representing a mean of 68% of a 500 m circular buffer centered on unlogged trap clusters and eucalypt regrowth (excludes riparian buffers) representing an average of 56% of the buffer surrounding regrowth trap clusters. One unlogged cluster sampled lower elevation forest, two regrowth clusters sampled mid‐elevation, while one regrowth and one unlogged cluster sampled higher elevations (Figure 1). Additional clusters were deployed later in the study and sampled low‐elevation regrowth and high‐elevation old growth.

We applied approved flanged‐metal bat bands from the Australian Bird and Bat Banding Scheme to the forearm of captured bats. Study protocols were approved by the Department of Primary Industry, Forestry Corporation NSW Animal Ethics Committee (Animal Research Authority: 12/17). Band injuries on recaptured bats were noted (0 = no injury, 1 = minor abrasion, 2 = minor swelling, and 3 = serious swelling of forearm requiring band removal). We recorded <1% of recaptures with a swelling that required us to shift the band to the other forearm, indicating that band injuries were rare (unlike split metal types, Baker et al., 2001). Bats were sexed, weighed, forearm lengths measured, and teeth wear noted. As epiphyses had typically fused by the time of Autumn trapping, we were unable to reliably identify juveniles by this method. Eight percent of bats were classified as juveniles (3–4 months old) at capture, but on some occasions, no captured bats were identified as juveniles, because of either lower survival or an earlier birth. We pooled all bats within species across sex and age class to simplify modeling and maximize data availability.

Research was undertaken with a NSW Department of Planning and Environment Scientific License (SL 100623).

### 2.3 Climatic variation

Annual climate in the study area was collated for comparison with longitudinal trends in bat density. Rainfall data were collected in gauges on‐site while maximum temperature was interpolated to the mid‐point of the study area using SILO data (https://www.longpaddock.qld.gov.au/silo/). Data were lagged by one‐year preceding trapping in March of each year.

### 2.4 Analyses

Spatial capture–recapture models were used to analyze the data. All analyses were performed using the “secr” package (v. 4.3.1, Efford, 2020) in R (v.4.0.2, R Core Team, 2020). Model selection was based on the Akaike information criterion (AIC), first selecting the functional form of the capture function and then exploring the influence of covariates on parameters. Three functional forms of capture function were investigated (e.g., a half normal, uniform, and the hazard rate). The capture function is parameterized by *g*0 (the probability of being detected if the animal's home range center is placed on a trap) and *σ* (the spatial scale parameter, i.e., decay in detection probability from an individual's activity center). In the case of the hazard rate function, an additional home range shape parameter, *z*, gives the detection function extra flexibility. Once the functional form was selected, the influence of explanatory variables on *g*0 and *σ* was explored (Table 1).

**TABLE 1:** Covariates, modeled parameters, and the biological rationale/reason for use in the spatial capture–recapture models (SCR) analysis of bat banding data.

Covariates	Definition	Modeled parameters	Biological rationale
Minimum temperature	Mean minimum temperature of trap nights (°C)	*g*0	To account for lower bat activity on cooler nights when there was often rain
Elevation (high vs. low)	High = ≥600 m Low = <600 m	Density, *g*0	Activity of these species varies with elevation (Law & Chidel, 2002). A categorical split was made because sites did not evenly sample a gradient in elevation.
Year‐linear	1999–2020	Density	To assess whether density differed among years as a linear trend
Year‐factor	1999–2020	Density, *g*0, *σ*	To assess whether density differed among years as a factor
Mean maximum temperature	Mean daily maximum temperature (°C). Lagged by one‐year preceding trapping in March	Density	Temperature may affect survival or fecundity via its influence on insect prey
Annual rainfall	Rainfall (mm). Lagged by one‐year preceding trapping in March	Density	Annual rainfall may affect survival or fecundity via its influence on insect prey
Forest disturbance	Disturbance history within a 5 km buffer surrounding each cluster centroid. Disturbance recorded as regrowth (15 years since timber harvesting at the start of the study) or mapped old growth/rainforest (Law et al., 2018)	Density	Past timber harvesting history may influence density due to changes in tree‐hollow abundance and/or vegetation structure

In addition to the familiar assumptions of standard capture–recapture (e.g., there is no tag loss and the marking process does not influence survival), conventional SCR also assumes that animal home ranges are distributed randomly in space, and the population is closed and detection events are independent. Depending on the time of year, bats are known to live in colonies which may violate assumptions of independent detection events, as well as home range centers being distributed randomly. However, our surveys were timed (in autumn) to coincide with a season during which bats congregate to a lesser extent than in the spring–summer maternity season, so this assumption is met. A closed population is also assumed, but the mobility of bats and the long‐time series suggests the population was open even though previous analyses confirm that a high proportion of bats are resident at the study site in autumn (Law et al., 2018). To meet the closed population assumption, the data for each year were treated as new or distinct from other years: Any bat caught in multiple years was regarded as distinct individuals across distinct years but not within years. Thus, the data were effectively analyzed as a time series of closed populations across years, with “year‐factor” as a covariate for density. While treating recaptures among years as new can lead to some bias toward higher density estimates, we recorded relatively low recapture rates between years (see Section 3).

From 2018 to 2020, we recorded recaptures at the trap level, in addition to the cluster level, and compared SCR model bat density predictions made using the trap‐level (2018–2020) and cluster‐level (1999–2020: trap‐level information was collapsed for the period 2018–2020) capture events. However, all confidence intervals overlapped between the two models, because mostly traps within a cluster were separated by small distances (<200 m) relative to a bat's home range. Therefore, only the cluster‐level models are presented here.

## 3 RESULTS

### 3.1 Climatic variation over the study period

**FIGURE 2:**
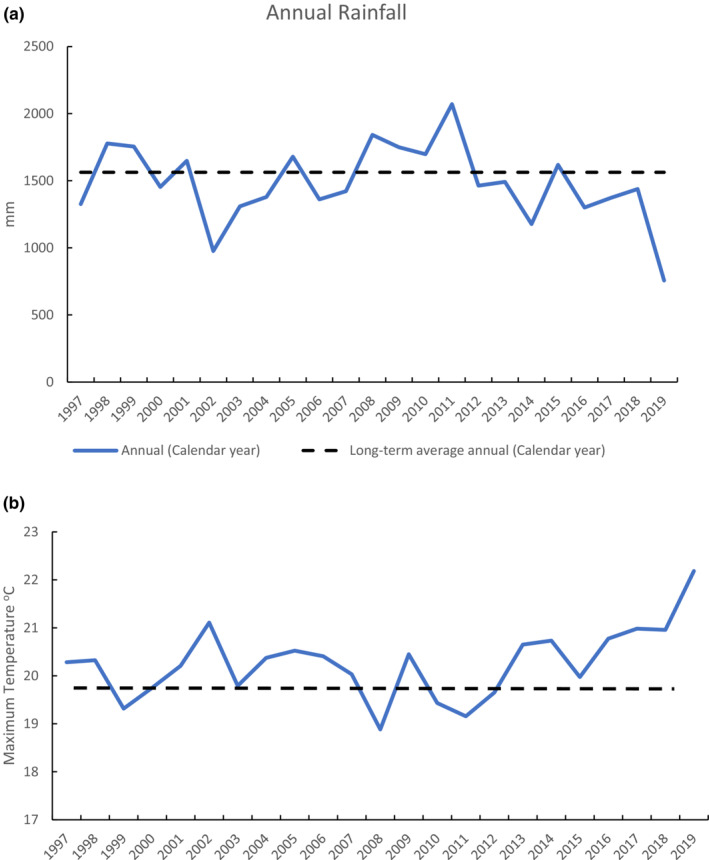
(a) Annual rainfall recorded at the Chichester State Forest study site over the trapping period, which began in 1999. Rainfall for the calendar year, which precedes trapping undertaken in March, is shown. (b) Mean daily maximum temperature in each year interpolated for the study area. Dashed lines show the SILO‐interpolated average since 1958.

The study area experiences high rainfall with a long‐term average (since 1958) just above 1500 mm. This varied over the study period but remained at ~1000 mm and 750 mm during drought years of 2002 and 2019, respectively (Figure 2). Conversely, rainfall peaked in 2011 during a La Nina period. The interpolated mean annual maximum temperature for the study area followed the inverse pattern of rainfall, with an increasing trend in temperature after 2012 (Figure 2).

### 3.2 Bat capture data

**FIGURE 3:**
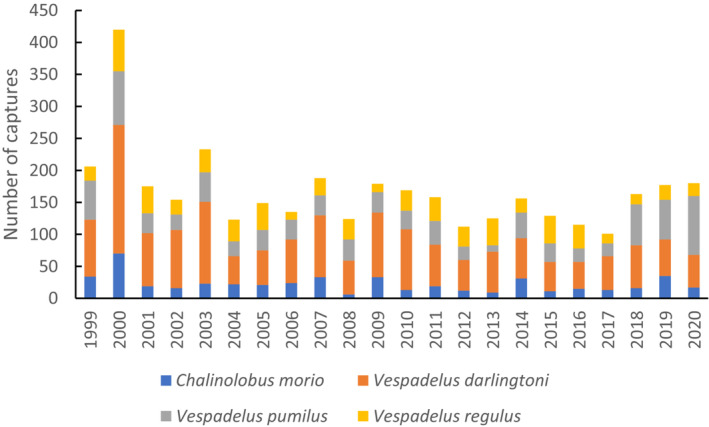
Total number of capture events of each species by year. Three trapping events were undertaken in the first 2 years of the study, after which there was a single trapping event per annum. An acoustic lure was used on some traps in 2016.

**FIGURE 4:**
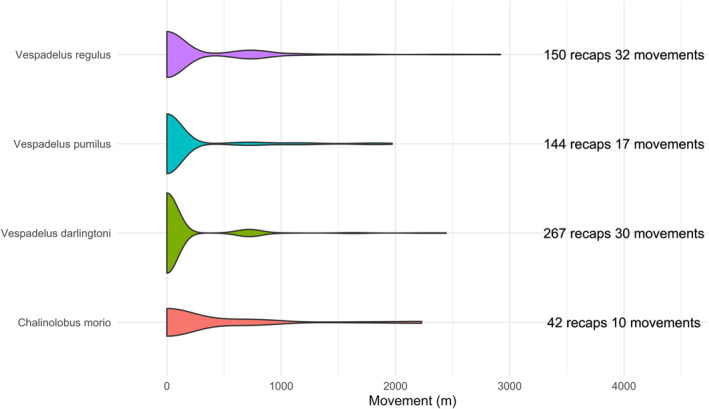
Violin plot of bat movement distance between clusters by species (1999–2020).

Across the entire data set (1999–2020), there were 3671 capture events of *V. pumilus*, *V. darlingtoni*, *V. regulus*, and *C. morio* (Figure 3). *Vespadelus darlingtoni* was the most frequently captured species (Figure 3). The peak in captures in 2000 was related to undertaking three rather than one trapping event. One of these was undertaken 1 month earlier (February) in that year compared with other years, coinciding with high numbers of juveniles that likely suffer high mortality in the subsequent month (Law et al., 2018). Recaptures represented 16% of all captures (603 out of 3671 capture events). Of these, 89 were between‐cluster movements (Figure 4). The maximum recapture distance between clusters was 2.8 km for *V. regulus*, 2.45 km for *V. darlingtoni*, 2.25 km for *C. morio*, and 1.99 km for *V. pumilus*.

Bats were typically caught just once, or if multiple times, typically in a single year only, but some individuals were recaptured over a 4‐ or 5‐year period. Some of these individuals were captured up to eight times and the longest time to recapture was 11 years for *V. darlingtoni*, 9 years for *V. regulus*, 7 years for *V. pumilus*, and 8 years for *C. morio*. If a bat was recaptured multiple times, it was typically captured at the same location regardless of whether it was captured within the same year or between years.

### 3.3 SCR model selection results

Model selection results for density are provided for the cluster‐level analysis over the full study period (Table 2; see Appendix S1 for full model results), and each species is considered separately below. Year as a linear trend was not supported in any of the selected models.

**TABLE 2:** Akaike information criterion (AIC) model selection results for the top three models for four bat species when analyzed to the cluster level (1999–2020).

Species	Model	npar	AIC	AICc	dAICc	AICcwt
*Vespadelus pumilus*	*D* ~ year‐factor + elevation *g*0 ~ elevation *σ* ~ 1 *z* ~ 1	27	1911.27	1913.48	0.00	0.60
	*D* ~ year‐factor + elevation *g*0 ~ 1 *σ* ~ 1 *z* ~ 1	26	1912.22	1914.28	0.79	0.40
	*D* ~ year‐factor *g*0 ~ elevation *σ* ~ 1 *z* ~ 1	26	1945.60	1947.65	34.17	0.00
*Vespadelus darlingtoni*	*D* ~ year‐factor + elevation *g*0 ~ elevation *σ* ~ 1 *z* ~ 1	27	2809.51	2810.62	0.00	0.81
	*D* ~ year‐factor + elevation *g*0 ~ 1 *σ* ~ 1 *z* ~ 1	26	2812.49	2813.52	2.90	0.19
	*D* ~ year‐factor *g*0 ~ elevation *σ* ~ 1 *z* ~ 1	26	2829.67	2830.70	20.08	0.00
*Vespadelus regulus*	*D* ~ year‐factor *g*0 ~ elevation *σ* ~ 1 *z* ~ 1	26	1715.14	1718.01	0.00	0.74
	*D* ~ year‐factor + elevation *g*0 ~ elevation *σ* ~ 1 *z* ~ 1	27	1716.95	1720.05	2.04	0.26
	*D* ~ year‐factor + elevation *g*0 ~ 1 *σ* ~ 1 *z* ~ 1	26	1738.58	1741.46	23.45	0.00
*Chalinolobus morio*	*D* ~ year‐factor + elevation *g*0 ~ 1 *σ* ~ 1 *z* ~ 1	26	1436.68	1439.99	0.00	1.00
	*D* ~ year‐factor *g*0 ~ 1 *σ* ~ 1 *z* ~ 1	25	1449.29	1452.36	12.36	0.00
	*D* ~ forest.type + elevation *g*0 ~ 1 *σ* ~ 1 *z* ~ 1	6	1455.71	1455.90	15.90	0.00

*Note*: All models used a hazard rate detection function. *D* = density, *g*0 = detection probability, *σ* = spatial scale parameter, *z* is an additional shape parameter, and elevation is a factor variable (<600 m or ≥600 m).

### 3.4 Vespadelus pumilus

There were 586 unique *V. pumilus* banded across the study. The best model predicted density to vary with year factor and elevation (high/low), *g*0 with elevation, and other parameters constant (i.e., *σ* and *z*). On average, the species was estimated to have a higher detection probability at low elevation (*g*0 = 0.43 ± 0.06) compared with high elevation (*g*0 = 0.25 ± 0.04). The second model fell within two AIC, and it provided support for the same covariates, except *g*0 was constant. Annual maximum temperature was not supported as significantly influencing density across the study period and models with annual rainfall failed to converge.

**FIGURE 5:**
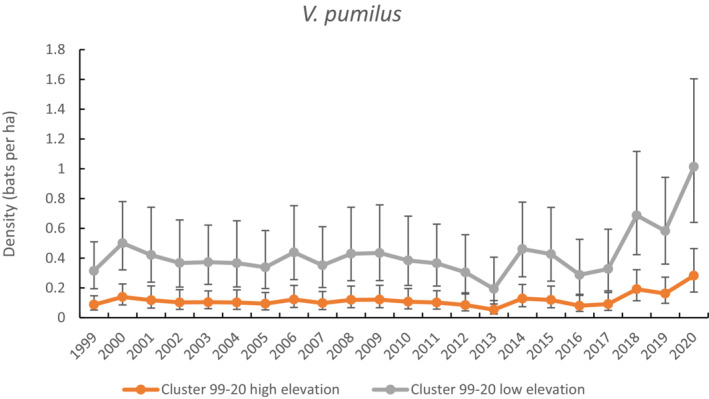
Comparison of density estimates for *V. pumilus* when capture data were limited to the cluster level (1999–2020). High elevation is >600 m, and low elevation is <600 m.

A plot of the density predictions for *V. pumilus* for the whole time series using the best‐fitting cluster‐level model shows density averaged four times higher at low elevation (0.43 bats ha^−1^) than at high elevation (0.12 bats ha^−1^) (Figure 5). Density was relatively stable across most of the time series, except for an increasing trend since 2013 to more than double the pre‐2013 average in 2020 coinciding with a period of increasing annual temperatures in the study area (Figure 2b).

### 3.5 Vespadelus darlingtoni

There were 1048 unique *V. darlingtoni* banded across the study. The best model allowed density to vary with year factor and elevation, *g*0 to vary with elevation, and other parameters to remain constant (i.e., *σ* and *z*). On average, for the cluster‐level model, the species was more likely to be detected at higher (>600 m) than lower elevation (<600 m) with an elevation‐specific *g*0 value estimated for low elevation (0.24 ± 0.04) and high elevation (0.37 ± 0.05). Annual maximum temperature was not supported as influencing density across the study period and models with annual rainfall failed to converge.

**FIGURE 6:**
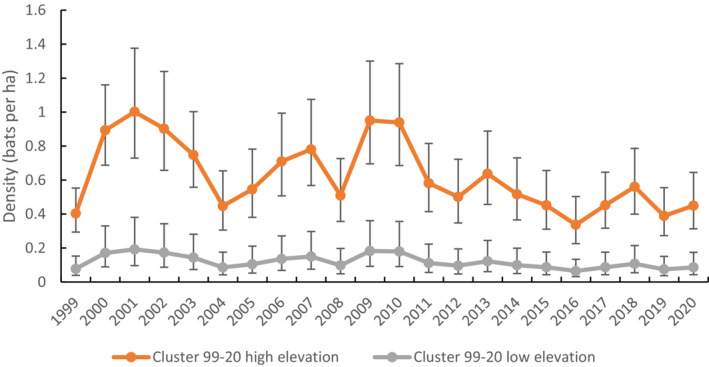
Comparison of density estimates for *Vespadelus darlingtoni* when capture data were limited to the cluster level (1999–2020). High elevation is >600 m, and low elevation is <600 m.

A plot of the density predictions of *V. darlingtoni* for the whole time series using the best‐fitting cluster‐level model shows density averaged more than five times higher at high elevation (0.62 bats ha^−1^) than at low elevation (0.12 bats ha^−1^) (Figure 6). Density fluctuated considerably across the time series, with a pattern for increasing density during years of mild, average to high rainfall (2000–2001 and 2009–2010) (Figure 2). No increasing trend was apparent after 2013.

### 3.6 Vespadelus regulus

There were 370 unique *V. regulus* banded across the study. The best model predicted density to vary with year factor and *g*0 with elevation while other parameters were constant (i.e., *σ* and *z*). On average, for the cluster‐level model, the species was more likely to be detected whether its activity center was at higher elevation (>600 m) than at lower elevation (<600 m) with an elevation‐specific *g*0 value estimated for low elevation (0.11 ± 0.03) and high elevation (0.78 ± 0.12). A second model within 2.04 AIC (26% weight; Table 2) indicates some support for density to increase with elevation. Annual maximum temperature was not supported as influencing density across the study period and models with annual rainfall failed to converge.

**FIGURE 7:**
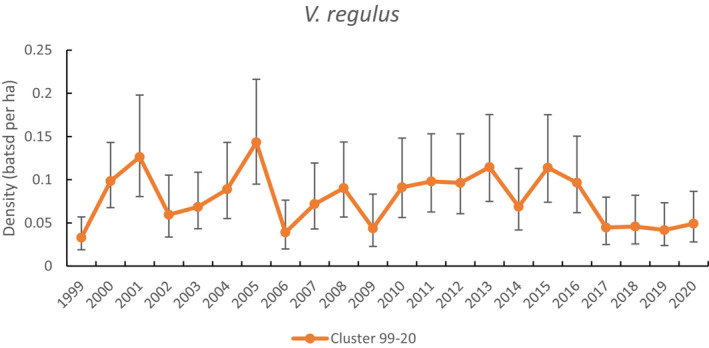
Comparison of density estimates for *Vespadelus regulus* when capture data were limited to the cluster level (1999–2020).

A plot of the density predictions for *V. regulus* for the whole time series using the best‐fitting cluster‐level model shows density averaged (0.08 bats ha^−1^) (Figure 7). Density fluctuated across the time series, with major dips associated with some hot, drought years (2002, 2006, and 2017–2020), a hot, wet year (2009), and a mild, wet year (Figure 2). Density remained low after 2017.

### 3.7 Chalinolobus morio

There were 364 unique *C. morio* banded across the study. The best model predicted density to vary with year factor and elevation, with other parameters constant (i.e., *g*0, *σ*, and *z*). On average, for the cluster‐level model, the species was estimated to have a low, but constant detection (*g*0 = 0.19, SE = 0.07). Annual maximum temperature was not supported as influencing density across the study period and models with annual rainfall failed to converge.

**FIGURE 8:**
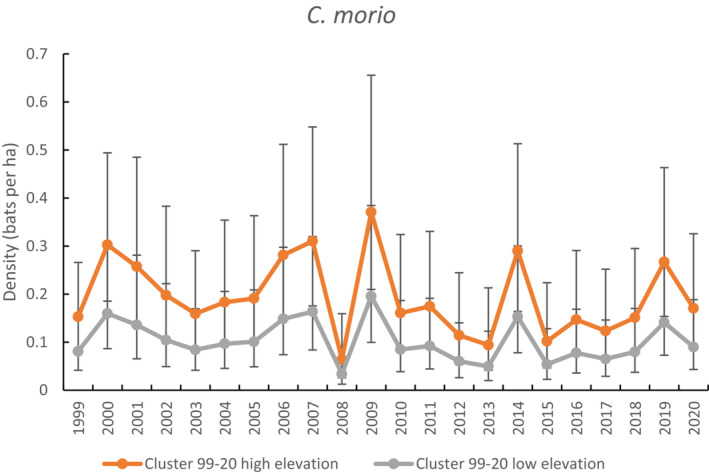
Comparison of density estimates for *Chalinolobus morio* when capture data were limited to the cluster level (1999–2020). High elevation is >600 m and low elevation is <600 m.

A plot of the density predictions for *C. morio* for the whole time series using the best‐fitting cluster‐level model shows density averaged almost two times higher at high elevation (0.19 bats ha^−1^) than low elevation (0.10 bats ha^−1^) (Figure 8). Density fluctuated across the time series, with a large dip in 2008, the coolest year of the study when annual rainfall was also above average. Density decreased by 50% between 2002 and 2005 (hot drought) and was also lower in 2013 (hot, average rainfall) and 2015–2018 (hot, below average rainfall), but then increased in 2019 (hot drought).

## 4 DISCUSSION

Despite deploying a trapping network comprised of only six to nine (three additional sites were added in 2013) permanent site clusters that were sampled annually, SCR modeling yielded plausible density estimates for each species. These results provide one of the first estimates of density for tree‐hollow roosting bats in large contiguous forests, and these were an order of magnitude larger than density estimates for bats in a recent global review (Santini et al., 2022). We acknowledge that our density estimates were associated with variable levels of precision that were largely determined by sample size of recaptures. A key feature of our study relates to the longevity of banding (>20 years), and of the bats themselves (up to ~10 years), as this combination provides opportunities for accumulating occasional recaptures between sites over time, especially when recapture rates are low. This, of course, violates the closure assumption of SCR modeling, but recaptures among years were few and we dealt with closure for each bat recaught in different years by treating them as a new individual. Nonetheless, we recommend that future modeling of bat density explores open population scenarios, although these should aim to increase recaptures among years potentially via increasing the number of trap locations and/or trapping effort. Other SCR studies have demonstrated reliable estimates of population density are possible for wide‐ranging animals and are relatively insensitive to the size of the trap array and trap dispersion provided it is similar to or larger than the extent of individual movements (Sollmann et al., 2012). In addition, reliable density estimates are possible where detection rates are low, though this comes at the cost of less precision (Carter et al., 2019).

We found that density varied substantially between species but not in relation to their body masses, with density at preferred elevations averaging 0.63 ha^−1^ for *V. darlingtoni* (6 g), 0.43 ha^−1^ for *V. pumilus* (4 g), 0.19 ha^−1^ for *C. morio* (8 g), and 0.08 ha^−1^ for *V. regulus* (5 g). Density estimates were comparable, but slightly lower than the density of female *V. pumilus* estimated elsewhere around maternity roosts (0.5–0.7 ha^−1^) based on roost emergence counts and estimated foraging distance (Law & Anderson, 2000), noting maternity roosts may be located in the most suitable locally available habitat. While our estimates are several times higher than the average identified for bats (typically <0.1 ha^−1^; Santini et al., 2022), there are few published studies. Our density estimates are an order of magnitude greater than that estimated in upland spruce forests of Germany (Milchram et al., 2020) and up to five times greater than recorded in northern England (Jones et al., 1996). While it is difficult to compare studies using different methods, we suspect the difference may in part be attributed to productivity at our study area, which experiences high rainfall and a mild climate with deeply dissected terrain and tall montane forest, though data on invertebrate numbers are currently lacking. Moreover, the study area is embedded within extensive forest that only experiences some anthropogenic disturbance in the form of regulated timber harvesting (see below) compared with extensive anthropogenic activity in Europe. In addition, our study species are some of the most commonly captured bats in their preferred forest habitat in south‐eastern Australia (Van Dyck & Strahan, 2008). A less commonly captured species, *Miniopterus orianae*, was estimated to have a lower density in south‐eastern Australia of 0.03 ha^−1^, based on roost counts and presumed foraging area (Dwyer, 1966). Previous acoustic surveys at our study area found relatively high bat activity along tracks, averaging 180–200 passes per night, around four times higher than forests elsewhere in the region (Lloyd et al., 2006) and ~ six times higher than urban areas in Australia (Threlfall et al., 2012), but around a third less than activity in small remnants of woodland on fertile agricultural land (Law & Chidel, 2006). In comparison with our estimates for bats, density of insectivorous birds in south‐eastern Australia is broadly comparable (<0.1 ha^−1^, Debus, 2006; 0.35–0.86 ha^−1^, Major et al., 1999). These densities are consistent with mammal and bird density being higher in temperate wet areas with intermediate levels of productivity (Santini et al., 2018).

At a broader level, variation in density was related to cluster elevation for most of the species modeled. Density was greater at higher elevations (up to 1000 m) for *V. darlingtoni*, *C. morio*, and as a minor effect for *V. regulus*, whereas, conversely, density was greater at low elevations for *V. pumilus*. These patterns are consistent with earlier analyses of population size from our banding data (Law et al., 2018) and call activity data for the study area (Law & Chidel, 2002), except that an elevation effect was not detected for *C. morio* in the former, probably because of low recapture rates for this species. A negative relationship with elevation for *V. pumilus* can be explained by the fact that it is a subtropical species and so warmer environments at lower elevations may be a physiological requirement, especially as the site occurs at the edge of its southern inland range (Law et al., 2008). Notably, it experiences low survival in our study area (Law et al., 2018). This may also explain the increase in *V. pumilus* density with the warming climate toward the end of our study (see below). Positive relationships with cooler, high elevations among the remaining species may reflect their more temperate, southern origins and also a productivity gradient with elevation in warmer months (Braithwaite, 1996) that can be exploited by species able to enter torpor when conditions are unsuitable for foraging (Turbill et al., 2003).

Elevation also influenced *g*0 (detection) for *V. darlingtoni* (+ve), *V. pumilus* (−ve), and *V. regulus* (+ve), though alternative models with constant *g*0 also had some support, suggesting this effect may not be strong. Better detection at certain elevations may be related to species‐specific activity levels, which varies with elevation at our study site (Law & Chidel, 2002) and likely leads to higher probability of capture. Notably, *g*0 was constant for *C. morio* and its activity does not vary with elevation at this site (Law & Chidel, 2002). Also, estimates of *g*0 may have been related to the flight patterns of each species as models estimated medium detection probability for *V. pumilus* (often low‐flying), low constant detection for *C. morio* (often higher‐flying), and intermediate values that increased with elevation for *V. darlingtoni* and *V. regulus*.

In contrast to elevation, we found no relationship between density and forest disturbance history. Resilience to forest disturbance is consistent with previous acoustic studies that have found edge‐space species frequently use tracks to avoid cluttered regrowth and hence tolerate past disturbance if suitable hollow trees are retained (Adams et al., 2009; Law & Chidel, 2002; Lloyd et al., 2006). Survival analyses at our site also found that the effect of disturbance history was species‐specific, with no detectable effect for two species (*V. darlingtoni* and *C. morio*), a positive effect for one (*V. pumilus*), and negative for the other (*V. regulus*), although overall the effects were minor (Law et al., 2018). *Vespadelus pumilus* forages, roosts, and breeds in regrowth‐dominated forests as well as old growth (Law & Anderson, 2000).

A key feature of our study was its longitudinal nature spanning 20 years through both wet and dry conditions and a warming climate due to global climate change (Lewis & King, 2015). Longitudinal studies of birds have shown rainfall, and the southern oscillation index, is correlated with breeding in temperate Australia (Marchant et al., 2016). In tropical Queensland, a 17‐year study found most montane bird specialists had undergone dramatic declines with a warming climate (Williams & de la Fuente, 2021). In our subtropical montane study, bat density (and precision) fluctuated over time, sometimes substantially. Annual maximum temperature was not supported as influencing density and models of annual rainfall failed to converge, which we suggest relates to the following potential factors: inconsistent relationships of density with complex climatic patterns over 20 years, effects of longer or shorter lags than we tested and/or insufficient data for adequate testing. Nonetheless, some patterns were evident that are suggestive of the importance of climatic conditions. Increases were associated with wet years (*V. darlingtoni*) and decreases in some hot, dry years (*V. regulus*) or hot years (*C. morio—*and the coolest year of the study). *Vespadelus pumilus* exhibited the most marked response with an increasing trend since 2013 that paralleled an increase in temperature in the study area. This subtropical species, which occurred at higher densities at low elevation, may benefit from a warming climate, whereas density of *V. regulus*, a cool temperate species, remained low after 2017 when temperatures peaked in our study. While these trends were mostly weak, our wet montane study area is a climate refuge, and so climate extremes over the course of the study were generally buffered (Law et al., 2018). Larger fluctuations in density might be expected in areas exposed to greater fluctuations in climate. Unfortunately, there are few long‐term datasets available for bats to test these ideas (Law & Blakey, 2021). Recent analysis of trends in ultrasonic activity over 5 years in dry forests and woodlands of Australia have revealed that large variations in activity were negatively related to preceding wet winters rather than drought (Law et al., 2021). We suspect recruitment rather than adult survival to be the more sensitive population attribute affected by climate conditions in south‐eastern Australia, as was found for the trawling large‐footed myotis *Myotis macropus* (Law et al., 2020). Recruitment may have greater influence on density than survival for insectivorous bats because torpor is used to save energy during times of environmental stress (Geiser & Turbill, 2009).

In summary, our results suggest that bat densities in Australian forests may be higher than previously appreciated, which would reinforce their perceived importance in providing ecosystem services. More studies are needed in different habitats and continents to place the densities we estimated into a broader context. For example, the effects of climate change on trends in density are expected to be greater where drought effects are more pronounced than in our climate refugia.

## AUTHOR CONTRIBUTIONS

**Bradley Law:** Conceptualization (lead); funding acquisition (lead); methodology (lead); project administration (lead); writing – original draft (lead); writing – review and editing (lead). **Traecey Brassil:** Data curation (lead); formal analysis (supporting); investigation (supporting); project administration (supporting); writing – original draft (supporting); writing – review and editing (supporting). **Roland Proud:** Formal analysis (equal); software (equal); writing – original draft (supporting). **Joanne Potts:** Formal analysis (equal); software (equal); writing – original draft (supporting).

## CONFLICT OF INTEREST STATEMENT

The authors declare no conflict of interest.

## FUNDING INFORMATION

Funding over the course of the study was provided by the NSW Government.

## Supporting information


Appendix S1

## Data Availability

Data are available from the Australian Bird and Bat Banding Scheme https://www.dcceew.gov.au/science‐research/bird‐bat‐banding.
