# Supplementary material for: Estimating density of forest bats and their long‐term trends in a climate refuge
Source: Ecol Evol. 2023 Jun 17;13(6):e10215. doi: 10.1002/ece3.10215 (PMC10276525; doi:10.1002/ece3.10215)
Supplement: Supplementary file 1 — Appendix S1 [file ECE3-13-e10215-s001.docx]

**Estimating density of forest bats and long-term trends in a climate refuge**

**Law, B., Brassil T. , Proud, R. and Potts, J.**

**Full model section results for each species**

Table 1: AIC model selection results for Vespadelus pumilus data when analysed to the cluster level (1999-2020).

| **Model** | **detectfn** | **npar** | **AIC** | **AICc** | **dAICc** | **AICcwt** |
| --- | --- | --- | --- | --- | --- | --- |
| D~year-factor + elevation g0~elevation sigma~1 z~1 | hazard rate | 27 | 1,911.27 | 1,913.48 | 0.00 | 0.60 |
| D~year-factor + elevation g0~1 sigma~1 z~1 | hazard rate | 26 | 1,912.22 | 1,914.28 | 0.79 | 0.40 |
| D~year-factor g0~elevation sigma~1 z~1 | hazard rate | 26 | 1,945.60 | 1,947.65 | 34.17 | 0.00 |
| D~forest.dist + elevation g0~1 sigma~1 z~1 | hazard rate | 6 | 1,949.63 | 1,949.75 | 36.26 | 0.00 |
| D~elevation g0~1 sigma~1 z~1 | hazard rate | 5 | 1,955.78 | 1,955.86 | 42.38 | 0.00 |
| D~1 g0~elevation sigma~1 z~1 | hazard rate | 5 | 1,993.26 | 1,993.34 | 79.86 | 0.00 |
| D~year-factor g0~1 sigma~1 z~1 | hazard rate | 25 | 2,060.99 | 2,062.89 | 149.41 | 0.00 |
| D~year-factor g0~year-factor sigma~1 z~1 | hazard rate | 46 | 2,064.74 | 2,071.26 | 157.78 | 0.00 |
| D~maxT g0~1 sigma~1 z~1 | hazard rate | 5 | 2,080.61 | 2,080.70 | 167.21 | 0.00 |
| D~year-factor g0~1 sigma~year-factor z~1 | hazard rate | 46 | 2,083.55 | 2,090.08 | 176.59 | 0.00 |
| D~Year-factor g0~1 sigma~1 z~1 | hazard rate | 5 | 2,098.77 | 2,098.86 | 185.37 | 0.00 |
| D~forest.dist g0~1 sigma~1 z~1 | hazard rate | 5 | 2,105.58 | 2,105.66 | 192.18 | 0.00 |
| D~1 g0~1 sigma~1 z~1 | hazard rate | 4 | 2,113.96 | 2,114.01 | 200.53 | 0.00 |

Table 2: AIC model selection results for Vespadelus darlingtoni data when analysed to the cluster level (1999-2020).

| **model** | **detectfn** | **npar** | **logLik** | **AIC** | **AICc** | **dAICc** | **AICcwt** |
| --- | --- | --- | --- | --- | --- | --- | --- |
| D~year-factor + elevation g0~elevation sigma~1 z~1 | hazard rate | 27 | -1,377.757 | 2,809.51 | 2,810.62 | 0.00 | 0.81 |
| D~year-factor + elevation g0~1 sigma~1 z~1 | hazard rate | 26 | -1,380.245 | 2,812.49 | 2,813.58 | 2.90 | 0.19 |
| D~year-factor g0~elevation sigma~1 z~1 | hazard rate | 26 | -1,388.834 | 2,829.67 | 2,830.70 | 20.08 | 0.00 |
| D~forest.dist + elevation g0~1 sigma~1 z~1 | hazard rate | 6 | -1,451.375 | 2,914.75 | 2,914.81 | 104.19 | 0.00 |
| D~elevation g0~1 sigma~1 z~1 | hazard rate | 5 | -1,452.914 | 2,915.83 | 2,915.87 | 105.25 | 0.00 |
| D~1 g0~elevation sigma~1 z~1 | hazard rate | 5 | -1,461.083 | 2,932.16 | 2,932.21 | 121.59 | 0.00 |
| D~year-factor g0~1 sigma~1 z~1 | hazard rate | 25 | -1,596.701 | 3,243.40 | 3,244.35 | 433.73 | 0.00 |
| D~year-factor g0~min_temp sigma~1 z~1 | hazard rate | 26 | -1,598.342 | 3,248.68 | 3,249.71 | 439.09 | 0.00 |
| D~forest.dist g0~1 sigma~1 z~1 | hazard rate | 5 | -1,624.237 | 3,258.47 | 3,258.52 | 447.90 | 0.00 |
| D~Year-factor g0~1 sigma~1 z~1 | hazard rate | 5 | -1,652.060 | 3,314.12 | 3,314.16 | 503.54 | 0.00 |
| D~maxT g0~1 sigma~1 z~1 | hazard rate | 5 | -1,655.605 | 3,321.21 | 3,321.254 | 510.633 | 0.0000 |
| D~1 g0~1 sigma~1 z~1 | hazard rate | 4 | -1,666.656 | 3,341.31 | 3,341.340 | 530.719 | 0.0000 |

Table 3: AIC model selection results for Vespadelus regulus data when analysed to the cluster level (1999-2020).

| **model** | **detectfn** | **npar** | **logLik** | **AIC** | **AICc** | **dAICc** | **AICcwt** |
| --- | --- | --- | --- | --- | --- | --- | --- |
| D~year-factor g0~elevation sigma~1 z~1 | hazard rate | 26 | -831.5678 | 1,715.14 | 1,718.01 | 0.00 | 0.735 |
| D~year-factor + elevation g0~elevation sigma~1 z~1 | hazard rate | 27 | -831.4747 | 1,716.94 | 1,720.05 | 2.04 | 0.265 |
| D~year-factor + elevation g0~1 sigma~1 z~1 | hazard rate | 26 | -843.2919 | 1,738.58 | 1,741.46 | 23.45 | 0.000 |
| D~1 g0~elevation sigma~1 z~1 | hazard rate | 5 | -873.2180 | 1,756.44 | 1,756.55 | 38.54 | 0.000 |
| D~elevation g0~1 sigma~1 z~1 | hazard rate | 5 | -884.2725 | 1,778.54 | 1,778.66 | 60.65 | 0.000 |
| D~forest.dist + elevation g0~1 sigma~1 z~1 | hazard rate | 6 | -884.1909 | 1,780.38 | 1,780.55 | 62.53 | 0.000 |
| D~year-factor g0~1 sigma~1 z~1 | hazard rate | 25 | -931.4645 | 1,912.93 | 1,915.59 | 197.57 | 0.000 |
| D~maxT g0~1 sigma~1 z~1 | hazard rate | 5 | -965.8523 | 1,941.71 | 1,941.82 | 223.81 | 0.000 |
| D~forest.dist g0~1 sigma~1 z~1 | hazard rate | 5 | -966.9251 | 1,943.85 | 1,943.97 | 225.96 | 0.000 |
| D~1 g0~1 sigma~1 z~1 | hazard rate | 4 | -973.6890 | 1,955.38 | 1,955.46 | 237.44 | 0.000 |
| D~Year-factor g0~1 sigma~1 z~1 | hazard rate | 5 | -972.9915 | 1,955.98 | 1,956.10 | 238.09 | 0.000 |

Table 4: AIC model selection results for Chalinolobus morio data when analysed to the cluster level (1999-2020).

| **Model** | **detectfn** | **npar** | **logLik** | **AIC** | **AICc** | **dAICc** | **AICcwt** |
| --- | --- | --- | --- | --- | --- | --- | --- |
| D~year-factor + elevation g0~1 sigma~1 z~1 | hazard rate | 26 | -692.338 | 1,436.68 | 1,439.995 | 0.00 | 1 |
| D~year-factor g0~1 sigma~1 z~1 | hazard rate | 25 | -699.645 | 1,449.29 | 1,452.356 | 12.36 | 0 |
| D~forest.dist + elevation g0~1 sigma~1 z~1 | hazard rate | 6 | -721.854 | 1,455.71 | 1,455.897 | 15.90 | 0 |
| D~forest.dist g0~1 sigma~1 z~1 | hazard rate | 5 | -724.750 | 1,459.50 | 1,459.635 | 19.64 | 0 |
| D~elevation g0~1 sigma~1 z~1 | hazard rate | 5 | -727.921 | 1,465.84 | 1,465.978 | 25.98 | 0 |
| D~maxT g0~1 sigma~1 z~1 | hazard rate | 5 | -731.871 | 1,473.74 | 1,473.878 | 33.88 | 0 |
| D~Year-factor g0~1 sigma~1 z~1 | hazard rate | 5 | -732.239 | 1,474.48 | 1,474.614 | 34.62 | 0 |
| D~1 g0~1 sigma~1 z~1 | hazard rate | 4 | -733.825 | 1,475.65 | 1,475.740 | 35.75 | 0 |
| D~year-factor g0~elevation sigma~1 z~1 | hazard rate | 26 | -711.466 | 1,474.93 | 1,478.251 | 38.26 | 0 |
| D~1 g0~1 sigma~1 | halfnormal | 3 | -743.534 | 1,493.07 | 1,493.122 | 53.13 | 0 |
| D~1 g0~elevation sigma~1 z~1 | hazard rate | 5 | -746.884 | 1,503.77 | 1,503.904 | 63.91 | 0 |
